# *De Novo* Genome Sequence of a *Fusarium xylarioides* Race Pathogenic to Robusta Coffee (*Coffea canephora*) in Uganda

**DOI:** 10.1128/MRA.00520-19

**Published:** 2019-08-01

**Authors:** Sammy Olal, Daphne N. Bitalo, Nicholas D. Olango, Julius Mulindwa, Silver Ochwo, Stephen Obol Opiyo, Geoffrey Arinaitwe, Emmanuel Ogwok

**Affiliations:** a National Agricultural Research Organization, National Coffee Research Institute, Mukono, Uganda; b College of Veterinary Medicine, Animal Resources and Biosecurity, Makerere University, Kampala, Uganda; c Molecular and Cellular Imaging Center-Columbus OARDC, The Ohio State University, Columbus, Ohio, USA; Vanderbilt University

## Abstract

Here, we report the first whole-genome assembly of a *Fusarium xylarioides* race pathogenic to robusta coffee in Uganda. It comprises 55,122,624 bases and 14,552 genes. Gene ontology analysis assigned 5,720 genes to biological processes, 4,545 genes to cellular components, and 6,021 genes to molecular function.

## ANNOUNCEMENT

In Africa, coffee wilt disease (CWD) is endemic and remains an ongoing threat to coffee production (1). In Uganda, CWD causes an annual economic loss of $17 million (2). The disease is caused by a vascular fungal pathogen, Fusarium xylarioides. The pathogen has two races, each specific to a particular coffee species. Race 1 is pathogenic to robusta coffee (Coffea canephora), whereas race 2 is pathogenic to arabica coffee (Coffea arabica) (3). *F. xylarioides* causes coffee plants to wilt to mortality without recovering. We hereby announce the first whole-genome assembly of an *F. xylarioides* race pathogenic to robusta coffee in Uganda.

The culture used for sequencing was isolated from a CWD-affected robusta coffee plant in Uganda, previously deposited as a culture collection at CABI Bioscience in Wallingford, England (IMI number 379925). The fungus was grown on potato dextrose agar (CMO 139; Oxoid, England) for 5 days under 12-hour light and dark cycles at room temperature. DNA was extracted from mycelia using the plant/seed DNA miniprep kit (Zymo Research, USA) according to the manufacturer’s instructions. The purity and quantity of extracted DNA were prechecked using a spectrophotometer (Nanodrop 2000C; Thermo Scientific). The paired-end sequencing library was prepared using the TruSeq Nano DNA library prep kit. Quantity and quality of the library were checked on the Bioanalyzer 2100 (Agilent Technologies) using a high-sensitivity (HS) DNA chip, following the manufacturer’s instructions. The library was sequenced on the HiSeq 2500 Illumina platform (2 × 150-bp chemistry) to generate 10 Gb of data (67,472,516 reads). Adapter sequences were removed using Trimmomatic v0.32 (4) at a Phred score of ≥20. FastQC v0.11.5 (https://www.bioinformatics.babraham.ac.uk/projects/fastqc/) was used to check the quality of the reads.

*De novo* assembly of high-quality reads was accomplished using the SOAPdenovo v2.0 assembler (5). Gaps in assembled reads were closed using GapCloser v1.12, yielding a genome size of 55,122,624 bases without gaps and a G+C content of 34.37%. There were 1,521 contigs and 1,132 scaffolds of 55 Mb and an *N*_50_ value of 138,098 bp. The statistical elements of the assembly were calculated using sanger pathogen assemblystats v3.0 (https://github.com/sanger-pathogens/assembly-stats), an open-source program.

Genome assembly was assessed using the benchmarking universal single-copy orthologs (BUSCO) v3.0.2 software package (https://busco.ezlab.org/) from the OrthoDBv9 database (6) on genome mode, and it showed that the genome was 99.3% complete.

### Data availability.

This whole-genome shotgun project has been deposited at DDBJ/ENA/GenBank under the accession number RXHO00000000. The version described in this paper is the first version, RXHO01000000. The raw data have been deposited in the SRA under BioProject accession number PRJNA508603 and SRA accession number PRJNA508603.

## References

[1] Phiri N, Baker P 2009 A synthesis of the work of the Regional Coffee Wilt Programme 2000–2007. Coffee Wilt Disease in Africa. CABI, Wallingford, United Kingdom.

[2] Olal S, Olango N, Kiggundu A, Ochwo S, Adriko J, 2018 Using translation elongation factor gene to specifically detect and diagnose *Fusarium xylaroides*, a causative agent of coffee wilt disease in Ethiopia, East and Central Africa. J Plant Pathol Microbiol 9:440. doi:10.4172/2157-7471.1000440.

[3] Adugna G, Hindorf H, Steiner U, Nirenberg HI, Dehne HW, Schellander K 2005 Genetic diversity in the coffee wilt pathogen (Gibberella xylarioides) populations: differentiation by host specialization and RAPD analysis. J Plant Dis Protect 112:134–145.

[4] Bolger AM, Lohse M, Usadel B 2014 Trimmomatic: a flexible trimmer for Illumina sequence data. Bioinformatics 30:2114–2120. doi:10.1093/bioinformatics/btu170. 24695404 PMC4103590

[5] Luo R, Liu B, Xie Y, Li Z, Huang W, Yuan J, He G, Chen Y, Pan Q, Liu Y, Tang J, Wu G, Zhang H, Shi Y, Liu Y, Yu C, Wang B, Lu Y, Han C, Cheung DW, Yiu SM, Peng S, Xiaoqian Z, Liu G, Liao X, Li Y, Yang H, Wang J, Lam TW, Wang J 2012 SOAPdenovo2: an empirically improved memory-efficient short-read de novo assembler. GigaScience 1:18. doi:10.1186/2047-217X-1-18. 23587118 PMC3626529

[6] Waterhouse RM, Seppey M, Simão FA, Manni M, Ioannidis P, Klioutchnikov G, Kriventseva EV, Zdobnov EM 2018 BUSCO applications from quality assessments to gene prediction and phylogenomics. Mol Biol Evol 35:543–548. doi:10.1093/molbev/msx319. PMC5850278 29220515

